# Equations based on body mass index for body composition estimations of women presenting grade III obesity

**DOI:** 10.15446/rsap.V25n2.105516

**Published:** 2023-03-01

**Authors:** João Felipe Machado, Flávia Lúcia Conceição, João Régis Carneiro, Valéria Bender Bráulio, José Fernandes Filho

**Affiliations:** 1 JM: Educação Física. Ph.D. Ciências. Laboratório de Biociência, Movimento Humano (LABIMH), Hospital Universitário Clementino Fraga Filho, Universidade Federal do Rio de Janeiro (UFRJ). Rio de Janeiro, Brasil. prof.jfmachado@gmail.com Universidade Federal do Rio de Janeiro Movimento Humano (LABIMH) Hospital Universitário Clementino Fraga Filho Universidade Federal do Rio de Janeiro Rio de Janeiro Brazil; 2 FC: MD. Endocrinology. Ph.D. Medicina Endocrinologia. Hospital Universitário Clementino Fraga Filho, Universidade Federal do Rio de Janeiro (UFRJ). Rio de Janeiro, Brasil. flavia_lucia@yahoo.com.br Universidade Federal do Rio de Janeiro Medicina Endocrinologia Hospital Universitário Clementino Fraga Filho Universidade Federal do Rio de Janeiro Rio de Janeiro Brazil; 3 JC: MD. End. Ph.D. Medicina Clínica Médica. Hospital Universitário Clementino Fraga Filho, Universidade Federal do Rio de Janeiro (UFRJ). Rio de Janeiro, Brasil. endoregis.carneiro@uol.com.br Universidade Federal do Rio de Janeiro Medicina Clínica Médica Hospital Universitário Clementino Fraga Filho Universidade Federal do Rio de Janeiro Rio de Janeiro Brazil; 4 VB: MD. Ph.D. Graduado Medicina. Clínica Médica. Hospital Universitário Clementino Fraga Filho, Universidade Federal do Rio de Janeiro (UFRJ). Rio de Janeiro, Brasil. vbender2001@yahoo.com.br Universidade Federal do Rio de Janeiro Clínica Médica Hospital Universitário Clementino Fraga Filho Universidade Federal do Rio de Janeiro Rio de Janeiro Brazil; 5 JF: Educação Física. Ph.D. Genética Aplicada ao Esporte. Ph.D. Laboratório de Biociência do Movimento Humano (LABIMH), Hospital Universitário Clementino Fraga Filho, Universidade Federal do Rio de Janeiro (UFRJ). Rio de Janeiro, Brasil/Centro de Excelência em Avaliação Física (CEAF). Rio de Janeiro, Brasil. jff@ceafbr.com.br Universidade Federal do Rio de Janeiro Laboratório de Biociência do Movimento Humano Hospital Universitário Clementino Fraga Filho Universidade Federal do Rio de Janeiro Rio de Janeiro Brazil

**Keywords:** Severe obesity, body mass index, body composition, bioimpedance *(source: MeSH, NLM)*, Obesidad mórbida, índice de masa corporal (IMC), composición corporal, bioimpendacia, ecuaciones-métodos *(fuente: DeCS, BIREME)*

## Abstract

**Objective:**

To develop and validate predictive equations to estimate the body composition of women with grade III obesity, using the body mass index (BMI) as a predictive variable.

**Methods:**

This cross-sectional study involved 104 patients treated at the hospital of the Universidade Federal do Rio de Janeiro randomly divided into two groups, the Equation Group, used to generate regression equations, and the Validation Group, used to validate the equations. Body fat mass (BFM), body fat percentage (BFP), skeletal muscle mass (SMM), fat-free mass (FFM) and total body water content (TBW) were valuated employing the bioimpedance method (InBody® 230).

**Results:**

Polynomial equations exhibited the best fit and a general trend of results normalized by height squared presenting higher coefficients of determination (r^2^) was noted, positively affecting equation validations. Only one exception was observed, since the body fat percentage index (BFPI) resulted in an even lower correlation with BMI. Only these variables exhibited low r^2^ (0.11 to 0.29), while r^2^ values ranged from 0.51 to 0.94 for the other results.

**Conclusion:**

Except for the BFP and BFPI, body composition can be estimated by the application of predictive BMI-based models. The equations employed for the indices normalized by the square of height were better predictors, while the use of equations that do not employ this normalization should consider the caveat that individuals with extreme BMI values (40 to 76 kg/m^2^) present greater estimate deviations in relation to the measured values.

## RESUMEN

**Objetivo:**

Desarrollar y validar ecuaciones predictivas para estimar la composición corporal de mujeres con obesidad III, utilizando el índice de masa corporal (IMC) como variable predictiva.

**Métodos:**

Este estudio transversal involucró a 104 pacientes atendidos por el Hospital Universitario de la Universidad Federal de Río de Janeiro, divididos aleatoriamente en dos grupos. La masa de grasa corporal (MGC), el porcentaje de grasa corporal (PGC), la masa musculoesquelética (MME), la masa libre de grasa (MLG) y el contenido de agua total (ACT) fueron valorados por el método de bioimpedancia (InBody® 230).

**Resultados:**

Las ecuaciones polinómicas presentaron un mejor ajuste y se observó una tendencia general de resultados normalizados, con mayores coeficientes de determinación (r^2^), lo cual afectó positivamente las validaciones de las ecuaciones. Se observó apenas una excepción, en relación con el PGC, pues el índice de porcentaje de grasa corporal (IPGC) tuvo una correlación menor con el IMC. Estas variables exhibieron un r^2^ bajo (0,11 a 0,29). Los valores de r^2^ oscilaron entre 0,51 y 0,94 para los demás resultados.

**Conclusión:**

Con excepción del PGC y el IPGC, la composición corporal puede estimarse por medio de la aplicación de modelos predictivos basados en el IMC. Las ecuaciones empleadas por los índices normalizados por el cuadrado de la estatura fueron mejores predictores, en tanto que el uso de las ecuaciones que no emplean esa normalización debe considerar la advertencia de que individuos con valores extremos de IMC (40 a 76 kg/m^2^) presentan una mayor estimación de las desviaciones en relación con los valores medidos.

Obesity is a chronic non-communicable disease (NCD) considered the most important nutritional disorder in both developing and developed countries 1. It is not a single disorder, but a heterogeneous group of conditions with multiple causes that ultimately result in the obesity phenotype 2 and is considered one of the most serious public health issues. Its prevalence has increased sharply in recent decades, which has led to a global obesity epidemic 3.

Epidemiological data regarding obesity have become alarming, whether in terms of increasing rates of prevalence and incidence, or in the implications related to associated diseases, also known as comorbidities 4. Due to higher incidence rates and a high risk factor, obesity is often associated to other metabolic and systemic comorbidities, such as primary hypertension and diabetes mellitus 1.

In this context, the body mass index (BMI) is universally accepted as an obesity indicator for the quantitative classification of obesity, as proposed by Quetelet (1835) 5. BMI has been widely applied because there is evidence of its use as a marker of mortality and metabolic changes 6,7. The World Health Organization (WHO) defines three levels of obesity severity in relation to health risk: Grade I obesity, with BMI values between 30 and 34.9 kg/m^2^; Grade II, with BMI values between 35 and 39.9 kg/m^2^; and Grade III (severe), with BMI values greater than 40 kg/m2. Two more levels were added to this classification by The American Society for Bariatric Surgery (ASBS), in order to adapt it to the severely obese population, namely super obesity, with BMI values between 50-60 kg/m2, and super/super obesity, with a BMI greater than 60 kg/m2 8. However, it has the drawback of not differentiating body fat mass from fat-free body mass. For example, very muscular individuals can be wrongly classified as obese, even with a low fat percentage, if the evaluator is not aware of this fact.

Several methods for assessing body composition are available, but even the simplest ones require specialized training 9, just as the equipment for the use of these methods is not always available. In addition to this limitation, although body composition assessment methods in obese individuals have been widely discussed, these evaluations are difficult in individuals with grade III obesity due to equipment limitations and the characteristics of the employed method. Some examples in this regard include the difficulty of the use of skinfold calipers due to the amplitude of skinfolds in obesity grade III, while methods such as such as DEXA, MRI and computed tomography are difficult to apply to patients with body mass above 150 kg.

According to Ling *et al.*^10^, bioimpedance is a valid tool for the assessment of total and segmental body composition in the general population. Excellent agreements for women have been reported in comparison with DEXA for whole body lean mass (0.95), fat mass (0.97) and body fat percentage (0.93). In this context, in addition to being widely applied to non-obese individuals 11-13, bioimpedance is a more accessible method for the evaluation of obese individuals, with none of the aforementioned operational limitations, as results are obtained through the resistance of an electric current to body tissues. Although the use of this technique is affected by clinical status (e.g., hydration, fluid retention, among others), Kyle *et al.*^14^ indicate the need for further studies employing this technique regarding populations displaying altered clinical status, including obesity.

Several efforts have been made to develop models to predict certain parameters like fat percentage from easily acquired data, such as the BMI 15-17, however, no studies specifically dedicated to the use of BMI to predict the body composition of women with grade III obesity are available.

According to Frankenfield *et al.*^18^, body composition assessment methods through the compartmentalized analysis of the total body mass allow for the determination of the different portions that each body tissue occupies in the body, offering more accurate results regarding each portion. In this context, this research aims to develop and validate equations to predict different body composition parameters in women with obesity grade III using BMI.

## METHODS

A cross-sectional design was applied in which adult female patients with grade III obesity were evaluated regarding their body composition using the bioimpedance technique (InBody® 230). Convenience sampling was applied, and the sample consisted of 104 patients under treatment for obesity, before undergoing bariatric surgery, at the Hospital Universitário Clementino Fraga Filho (HUCFF) from the Bariatric Surgery Program (PROCIBA) of the Universidade Federal do Rio de Janeiro (UFRJ). These patients were divided into two groups, the first formed by 62 participants whose data were used to generate the predictive equations, comprising the Equation Group (EG), and a second group formed by 42 participants, whose data were used to test and validate the equations, consisting of the Validation Group (VG).

The present study was approved by the HUCFF/ UFRJ Ethics and Research Committee, protocol CAAE: 38740314.4.0000.5257. The research was conducted in accordance with the principles of the Declaration of Helsinki, resolution 196/96 of the National Research Ethics Council and subsequent determinations in force.

Body composition assessments were performed using height measurements and the bioimpedance method. Height was measured using a Sanny^®^ brand stadiometer (0.1 cm precision).

Bioimpedance measures body composition by employing an electrical current. The bioimpedance assessment was performed herein by using an InBody^®^ 230 scale coupled to a multifrequency, segmental direct body composition analyzer and a tetrapolar system with eight electrodes (tactile electrodes). Multi-frequency bioimpedance (as applied herein) is considered more suitable for the assessment of obese individuals 19.

The following results obtained by the bioimpedance analysis were analyzed in this study: body mass (BM) in kg; body mass index (BMI) in kg/m^2^; body fat percentage (BFP) in %; fat body mass (FBM) in kg; skeletal muscle mass (SMM) in kg; fat-free mass (FFM) in kg and total body water content (TBW) in kg.

Considering previous evaluations that test the applicability of expressing the results of body composition in the form of indices normalized by the square of height 20,14, the values of the evaluated parameters were thus normalized in this way, generating the following indices: body fat percentage index (BFPI), fat body mass index (FBMI), skeletal muscle mass index (SMMI), fat-free mass index (FFMI), total body water index (TBWI).

### Statistical analyses

The results are presented through descriptive statistics (means, standard deviations, medians, minimum and maximum values). Nonlinear regression models were applied, due to a better fit (with a higher r^2^ value) and underwent a validation step based on comparisons between the EG and VG. Regression model validation allows for assessments on whether these predictive models perform well on data independent of adjusted models.

To compare the measured and calculated data from the regression models, the Shapiro-Wilk test was first used to test data normality, resulting in significant differences (p<0.05). Thus, nonparametric tests were used, and the Mann-Whitney test was used for mean comparisons. Subsequently, differences between measured and calculated data were evaluated using Bland-Altman diagrams 21, as well as regression analyses to evaluate the possible occurrence of proportion bias regarding the variability of these differences in relation to the means between measured and calculated results. In addition, a Student's t test was applied to assess whether the differences between measured and calculated results differed significantly from zero.

## RESULTS

The study sample consisted of 104 women with BMI values between 40 and 76.6 kg/m^2^ (average of 50.7 kg/m^2^) and ages ranging from 20 to 68 years old (average of 45.2 years). These patients were randomly divided into two groups for the validation treatment of the predictive equations. In addition to the absolute parameters, index calculations normalized by the square of height for each were also calculated. The Equation Group (EG) consisted of 62 women (ages 20 to 68), while the Validation Group (VG) comprised 42 women (ages 23 to 68).

Table 1 presents the descriptive statistical analyses for age, height and body composition, in relation to the total number of obese women sampled. As the application of data from a certain VG group can be limited to BMI values outside the range of variation of the EG values of the employed group, it is noteworthy that a similar BMI variation was observed between the two groups (Mann-Whitney test p=0.13), indicating this was not a concern for the present study. The VG group exhibited BMI ranging between 41.3 and 76.6 kg/m^2^, while the EG group presented BMI values ranging between 40.0 and 73.0 kg/m^2^.

**Table 1:** Descriptive statistics regarding body composition characteristics for the total number of individuals

GROUP GT (N= 104)	Mean	St Dev	Median	Minimum	Maximum
AGE (years)	45.2	11.7	46.0	20.0	68.0
HEIGHT (m)	1.61	0.06	1.61	1.50	1.78
BM (kg)	132.0	21.3	130.0	88.2	196.0
BMI (kg/m^2^)	50.7	7.61	48.5	40.0	76.6
BFPmed (%)	52.8	2.61	53.2	44.5	56.8
BFPcal (%)	52.9	1.54	52.9	49.6	54.9
BFPImed (%/m^2^)	20.4	2.06	20.4	15.8	24.8
BFPIcal (%/m^2^)	20.3	0.69	20.2	18.9	21.3
BFMmed (kg)	69.9	12.7	68.8	46.8	109.3
BFMcal (kg)	69.9	11.4	67.3	52.0	102.0
BFMImed (kg/m^2^)	26.8	4.83	26.0	18.0	42.7
BFMIcal (kg/m^2^)	26.9	4.69	25.8	19.7	40.8
SMMmed (kg)	35.2	5.95	34.2	22.8	52.6
SMMcal (kg)	35.1	4.51	33.6	29.9	53.5
SMMImed (kg/m^2^)	13.5	2.03	12.9	10.1	20.4
SMMIcal (kg/m^2^)	13.6	1.93	12.9	11.4	21.6
FFMmed (kg)	62.1	9.60	60.6	41.4	91.1
FFMcal (kg)	62.1	6.99	59.8	53.5	89.6
FFMImed (kg/m^2^)	23.8	3.20	23.0	18.4	34.5
FFMIcal (kg/m^2^)	23.8	2.97	22.8	20.3	35.8
TBWmed (kg)	46.1	7.33	44.7	30.9	67.3
TBWcal (kg)	46.2	5.64	44.4	39.5	68.7
TBWImed (kg/m^2^)	17.7	2.48	17.0	13.7	26.3
TBWIcal (kg/m^2^)	17.8	2.39	16.9	15.0	27.6

The evaluation of the applied regressions in relation to body composition as a function of BMI indicated that polynomial equations exhibited the best fit in relation to the EG. Table 2 displays each equation with its respective statistical parameters, namely the determination coefficient (r^2^), the adjusted coefficient of determination (r^2^adjust); and standard error of the estimate (SEE).

**Table 2:** Predictive equations derived from the EG group (n=62). The coefficient of determination (r^2^), adjusted coefficient of determination (r^2^adjust) and standard error of the estimate (SEE) are displayed

Equations	r^2^	r^2^ adjust	SEE
BFP = (-0.011 x BMI^2^) + (1.366 x BMI) + 12.650	0.29	0.26	2.45
BFPI = (-0.0037 x BMI^2^) + (0.494 x BMI) + 5.177	0.11	0.08	2.09
BFM = (-0.0152 x BMI^2^) + (3.144 x BMI) - 49.453	0.87	0.86	4.57
BFMI = (-0.0048 x BMI^2^) + (1.136 x BMI) - 18.064	0.94	0.94	1.23
SMM = (0.0073 x BMI^2^) - (0.207 x BMI) + 26.417	0.55	0.54	4.09
SMMI = (0.0035 x BMI^2^) - (0.135 x BMI) + 11.110	0.85	0.85	0.78
FFM = (0.0091 x BMI^2^) - (0.077 x BMI) + 42.037	0.51	0.50	6.86
FFMI = (0.0048 x BMI^2^) - (0.138 x BMI) + 18.08	0.85	0.85	1.24
TBW = (0.0081 x BMI^2^) - (0.145 x BMI) + 32.28	0.56	0.54	5.00
TBWI = (0.0041 x BMI^2^) - (0.147 x BMI) + 14.139	0.87	0.86	0.93

BFP value in %; BFPI in %/m^2^; BFM in kg; BFMI in kg/m^2^; SMM in kg; SMMI in kg/m^2^; FFM in kg; FFMI in kg/m^2^; TBW in kg; ICAT in kg/m^2^; MR in kcal; and MRI in kcal/m^2^.

**Figure 1:**
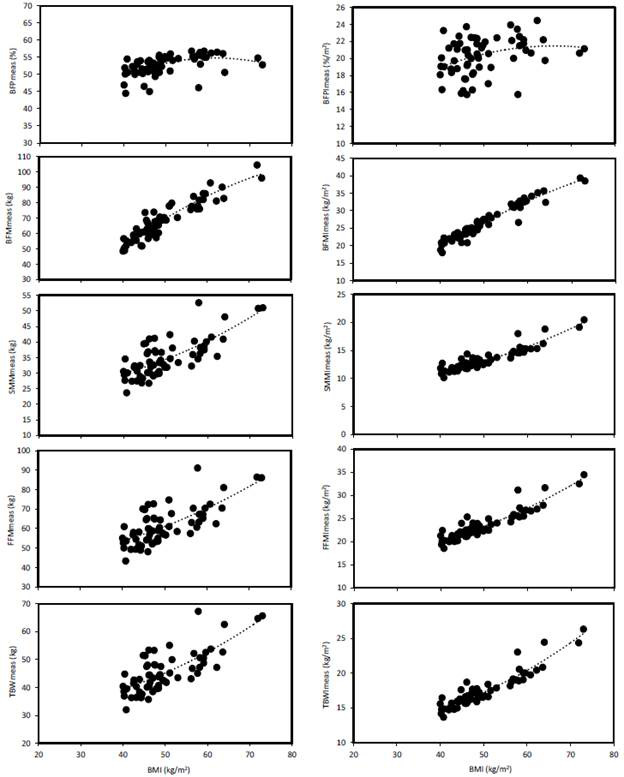
Regressions for body composition data as a function of the BMI

A general trend was observed for higher coefficients of determination and lower SEE values for height-normalized results, indicating that normalization positively affected equation validation. Only one exception was noted, in relation to the BFP, as the BFPI result in an even lower coefficient of determination (r^2^=0.n). However, the result is that low coefficients of determination were observed for the BFP and BFPI, not justifying their applicability, while all other tested variables exhibited high values (r^2^=0.51 to 0.94). Figure 1 demonstrates that these trends were consistent across the data sets and were not due to spurious correlations.

**Figure 2:**
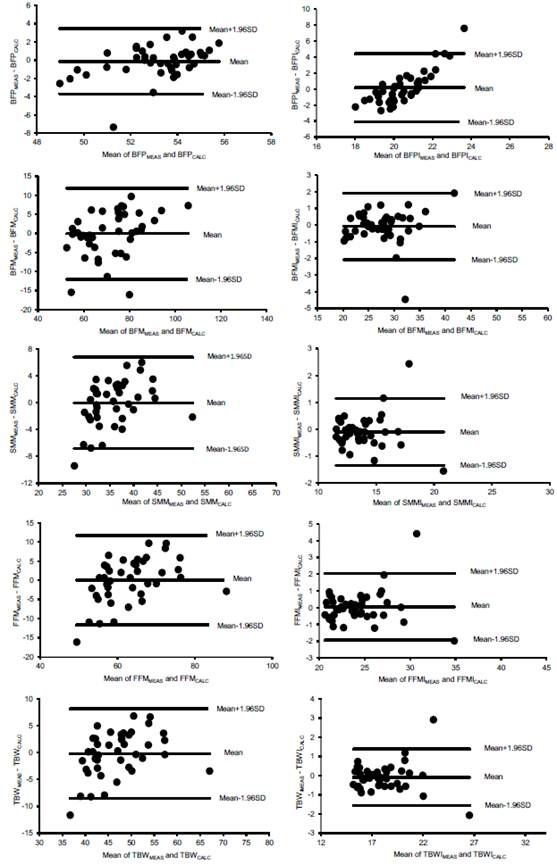
Bland-Altman plots for each evaluated body composition parameter

The Mann-Whitney test indicated no significant difference (p>0.05) between the measured and calculated regression results for all variables. The Figure 2 presents the plotted Bland-Altman graphs, indicating a comparatively low difference between measured and calculated data when compared to the mean results, although some exceptions were observed. The mean differences between measured and calculated data were not significantly different from zero according to the t test (p>0.05). On the other hand, simple linear regression tests between the differences between measured and calculated data (Table 3) indicate a bias, both above and below the means of the differences (p<0.05), in relation to the non-normalized data by the square of the height and for the BFPI. These observations confirm a generally greater applicability of predictive equations after normalization by the square of the height.

**Table 3:** Simple linear regressions used to assess whether differences between data measured and calculated by the predictive equations vary significantly as a function of the mean between these measured and calculated data (= proportion bias), as displayed in the Bland-Altman plots

Linear regressions	r^2^	P
BFPdif == -26.2563 + (0.4915 x PFBmean)	0.20	0.003
BFPIdif =-30.1419 + (1.4799 x IPGCmean)	0.70	0.000
BFMdif = -13.0999 + (0.1816 x MGCmean)	0.13	0.018
BFMIdif = -0.6601 + (0.0209 x IMGCmean)	0.01	0.539
SMMdif = -9.7646 + (0.2716 x MMEmean)	0.16	0.009
SMMIdif = -0.2385 + (0.0099 x IMMEmean)	0.001	0.846
FFMdif = -20.6922 + (0.3282 x MLGmean)	0.19	0.003
FFMIdif = -0.8826 + (0.0385 x IMLGmean)	0.01	0.457
TBWdif = -12.2569 + (0.2568 x ACTmean)	0.14	0.013
TBWIdif = -0.0823 - (0.0004 x IACTmean)	0.001	0.993

Significant results (p<0.05) are highlighted by p-values in bold.

All results except for the BFP and BFPI (i.e., low correlation values with the BMI), point to the validation of most of the predictive equations. However, the trend bias indicates that lower values tend to present a certain overestimation and higher values tend to present a certain underestimation of the data calculated by the regression models in relation to the means between measured and calculated data.

## DISCUSSION

The results of the present study reinforce a greater applicability of the BMI than has been explored in the literature regarding its application to predictive models on the body composition of large obese women. Furthermore, although the BMI does not exactly represent the body composition of individuals, it is easy to determine and a wide availability of data on body mass and height is available, which may be sufficient reasons to apply the BMI in epidemiological studies, in association or not with other anthropometric measurements 22.

The results also confirm that parameter normalization by the square of the height may comprise an even better option than that of non-normalized data, as indicated previously for non-obese individuals 20. This may be important when applied to different populations around the globe, indicating the need for further studies in this regard, especially for groups presenting more severe obesity, which are still scarcely studied in terms of prediction models based on the BMI.

There is, however, limited information on predictive equations for large obese subjects. Several previous studies that developed these kind of equations have included only a small number of large obese subjects in the populations sampled, usually evaluated within groups comprising predominantly non-obese individuals and those presenting less severe obesity 16,23,24. This is not interesting, as it has hindered understanding on how the very obese may require more specific predictive models for better treatment. For example, Jackson *et al.*^25^, evaluated only 10 women with a BMI between 40 and 50 kg/m^2^, to assess the effects of sex, age and race on the relationship between the BMI and BFP in a sample of 665 men and women, black and white, aged between 17 and 65 years old. The following nonlinear (polynomial) regression was obtained for the relationship between the BMI and BFP for women:

BFP = (4.3 5XBMI) - (0.05X35XBMI^2^) - 46.24 (r^2^=0.78, EPE =4.63 %)

However, literature findings indicate that the BMI and BFP ratio are not independent of age and sex, displaying a race effect for women but not for men. Failure to adjust for these sources of bias resulted in substantial differences in the proportion of individuals defined as obese by the BFP measured 25. Thus, these relationships must be interpreted cautiously.

It is worth noting that there is a temporal trend of increase in obesity grade III in the adult population of Brazilian capitals, with a greatest concern for women 26, requiring further epidemiological data. It is also recommended that more studies on predictive models be developed, for different populations from different regions.

It is also important to note that the findings reported herein for a female population in Rio de Janeiro indicates that the generated predictive models must be used cautiously, as:

1. The BFP and BFPI did not display good predictability compared to the other evaluated parameters;
2. A bias was observed in several cases, with higher and lower values being less accurate in terms of the predictive ability of the developed regression models.

The equations developed and validated herein serve as a contribution to the development and improvement of the knowledge about body composition. They are also of particular relevance due to their specificity for women with severe obesity, as obesity is currently one of the main diseases worldwide and is still difficult to treat and monitor. The lack of data on body composition and metabolic rate variables makes it difficult to advance in this regard, as current existing equipment is expensive and or not suitable for use by obese people presenting grade III obesity.

These results also contribute to meet the need to develop simple anthropometric nutritional status indicators that do not require a standard for comparison, in the sense of reflecting body composition, as highlighted previously 27.

The predictive equations available in the literature, which would be lower-cost methods, present some limitations as they were not created specifically for this high-obesity population. Therefore, our equations specifically created for women with grade III obesity comprise original contributions to help fill this worldwide gap, enabling a more accurate treatment and monitoring for this specific population.

In the context of obesity grade III, standardized and validated tools that measure functionality and health according to current WHO criteria and concepts are not available 28. This reinforces the demand for further validation studies on the body composition of individuals presenting this degree of obesity, as proposed in this study.

The bioimpedance technique employs different body characteristics that undergo several physical effects concerning hydration status, fat fraction, and body geometry on tissue conductivity, which in part explains why empirical predictive equation models are specific to certain populations 19. For example, Deurenberg 29 pointed out that, when evaluating individuals within the BMI classes from <18 to >3 5 kg/m^2^, bioimpedance presented greater uncertainties in more advanced obesity cases, due to the wide variability of the relationship between body composition and body bioimpedance, which may be due to relative increases in body water and extracellular water, leading to body fat underestimations and different body geometry leading to body fat overestimations. This explains why the BFP was not an adequate predictor in the present study.

Previous studies that developed BFP prediction models based on the BMI for both obese and non-obese individuals together indicate a slope in the BFM curves as a function of the BMI, where they lose the correlation trend as they reach obese BMI results 16,17,25.

The results confirm the possible limitation of the BMI for the prediction of body fat indicated for different populations, indicating low accuracy for grade III obese women, with r^2^ values (Table 2) explaining only 29 % and 11 % of the variability of BFM and BFMI results, respectively. On the other hand, a medium to high predictive capacity was observed in this application of the BMI for the other investigated body composition parameters, with r^2^ values explaining 51 % to 87 % of the data variability not normalized by the square of height. This prediction capacity was increased to 85 % to 94 % in relation to normalized data (except for the BFPI, as mentioned above). This corroborates the hypothesis that normalization can improve parameter prediction using BMI.

Bioimpedance can be used to monitor changes, diagnose deficiencies and formulate treatment recommendations in post-surgical assessments concerning how exercise positively influences body mass composition 30. In the case of the BFM, the use of predictive equations in expanding the possibilities of this type of assessment to more people with grade III obesity was not valid when employing the BMI, but the results indicate that this is possible for several other parameters.

On the other hand, Horie *et al.*^31^ concluded that, although standard bioimpedance technique equations developed for the general population are not accurate to assess the BFM in severely obese patients, the new equations developed for this population as a function of age, current weight and height are more accurate when compared to the original standard equation.

Thus, the results of the predictive equations generated in the present study (Table 2) corroborate that the BFM prediction can be adequately validated, as previously reported by Horie *et al.*^31^.

The BMI is widely used as a measure of overweight and obesity, but underestimates the prevalence of both conditions, defined as excess body fat, as pointed out by Gómez-Ambrosi *et al.*^32^. That study, which employed a total of 6 123 Caucasian individuals (924 thin, 1 637 overweight and 3 562 obese, classified according to their BMI), aged between 18 and 80, reported that 29 % of individuals classified as thin and 80 % of individuals classified as overweight according to their BMI exhibited BFPS within the obesity range. Considering the high cardiometabolic risk factors reported in non-obese individuals according to the BMI but obese based on body fat, the authors concluded that it is desirable to include body composition measures alongside morbidity assessments in everyday medical practice, both for diagnoses and for decision-making regarding the establishment of adequate obesity treatments.

Validated predictive equations can contribute to support obesity treatments, as evidenced in the scientific literature. For example, Azevedo *et al.*^33^, when evaluating the development of SMM values below estimated threshold, drew attention to the importance of long-term follow-up of patients undergoing bariatric surgery in order to obtain healthy weight loss. These authors suggested that further studies should include a higher number of patients and with longer follow-up periods, in order to implement specific interventions for patients undergoing bariatric surgery.

With the validation of SMM predictions from the BMI, monitoring the muscle mass maintenance in the postoperative period becomes more accessible and feasible. This study, thus, contributes in this sense for the SMM, as well as for the other evaluated parameters.

This development of the ability to predict the body composition of large obese women is particularly desirable, considering that obesity is one of the contemporary problems significantly affecting the social life and health of millions of Brazilian women, and that obesity grade III has led to increasing bariatric surgeries, especially among women 34.

It is clear that the pathophysiology of obesity can be associated to later comorbidities, such as SAH, diabetes mellitus and dyslipidemia. Besides that, non-communicable chronic diseases represent a significant burden for the public health system in Brazil, given that they are one of the main causes of death and illness in the Brazilian population. Due to its direct negative health effects, in addition to indirect effects resulting from associated chronic diseases, obesity, thus, represents a double burden on health systems 1.

In Brazil, the Ministry of Health, within the scope of the Unified Health System (SUS), through primary health care, is the main proposer of actions aimed at the prevention and treatment of obesity in recent years 35. Estimates of the costs attributable to the main chronic diseases associated with inadequate nutrition indicate the significant economic burden of these diseases for the SUS. The data indicate the need to prioritize integrated and intersectoral policies for the prevention and control of hypertension, diabetes and obesity 36.

Obesity is considered a global public health priority due to its magnitude and relationship with chronic diseases 37. Overweight and obesity in Brazil are important risk factors for hypertension and diabetes, coexisting in most diabetics and hypertensive patients. According to data from the 2013 National Health Survey (PNS), more than a third of diabetics and hypertensive patients were obese and 75 % of diabetics and 74 % of hypertensive patients were overweight 36.

Thus, adding obesity to these comorbidities allows for a more complete estimate of the economic impact of obesity on the SUS. With the incorporation of obesity costs as a risk factor for hypertension and diabetes, the total costs attributable to obesity increase to R$ 669 million in hospitalizations and outpatient expenses and to R$ 722 million in drug spending, totaling R$ 1.39 billion in 2018. Over 60 % of total expenditure attributable to obesity was with women, given the higher prevalence of obesity and the higher relative risk of some outcomes, particularly cardiovascular disease, in females 36.

This scenario increases the need to evaluate and propose safe and accurate, low cost and technically easy methods that can be widely employed by health professionals in the evaluation of individuals in health centers and clinics and in population studies, in order to ensure adequate targeting of intervention measures and health policies 38, as in the present study.

The present evaluation allowed the following conclusions regarding the application of predictive models for women with obesity grade III:

1. The results of the present study reinforce the wide applicability of the BMI, greater than what has been explored to date in the literature regarding its validity for use in predictive body composition models;
2. The equations developed for the BFP and the BFPI could not be validated, due to inadequate predictability to estimate their respective parameters;
3. All other equations developed from the use of indices were validated verified statistically, indicating that they can be used in the evaluation of women with obesity grade III;
4. The equations not developed using the indices were also statistically validated, indicating that they can be used in the assessment of women with grade III obesity. However, we must consider a caveat for this use, as a bias was observed in several cases, in which higher and lower values are less accurate in terms of the predictive capacity of the developed regression models.
